# Author Correction: The HSP90/R2TP assembly chaperone promotes cell proliferation in the intestinal epithelium

**DOI:** 10.1038/s41467-022-33519-y

**Published:** 2022-10-19

**Authors:** Chloé Maurizy, Claire Abeza, Bénédicte Lemmers, Monica Gabola, Ciro Longobardi, Valérie Pinet, Marina Ferrand, Conception Paul, Julie Bremond, Francina Langa, François Gerbe, Philippe Jay, Céline Verheggen, Nicola Tinari, Dominique Helmlinger, Rossano Lattanzio, Edouard Bertrand, Michael Hahne, Bérengère Pradet-Balade

**Affiliations:** 1 IGMM, Univ Montpellier, CNRS, Montpellier, France; 2 Equipe labélisée Ligue Nationale Contre le Cancer, Paris, France; 3 Centre d’Ingénierie Génétique Murine, Institut Pasteur, Paris, France; 4 IGF, Univ Montpellier, CNRS, INSERM, Montpellier, France; 5 Department of Medical, Oral and Biotechnological Sciences, Center for Advanced Studies and Technology (CAST), ‘G. d’Annunzio’ University of Chieti–Pescara, Chieti, Italy; 6 CRBM, Univ Montpellier, CNRS, Montpellier, France; 7 Department of Innovative Technologies in Medicine & Dentistry, Center for Advanced Studies and Technology (CAST), ‘G. d’Annunzio’ University of Chieti–Pescara, Chieti, Italy; 8 IGH, Univ Montpellier, CNRS, Montpellier, France

**Keywords:** Chaperones, Chaperones, Intestinal stem cells

Correction to: *Nature Communications* 10.1038/s41467-021-24792-4, published online 10 August 2021

In this article the grant number LS 139433-2016 relating to le comité Languedoc-Roussillon de la Ligue Contre le Cancer for Bérengère Pradet-Balade was omitted. The original article has been corrected.

