# Supplementary material for: The HSP90/R2TP assembly chaperone promotes cell proliferation in the intestinal epithelium
Source: Nat Commun. 2021 Aug 10;12:4810. doi: 10.1038/s41467-021-24792-4 (PMC8355188; doi:10.1038/s41467-021-24792-4)
Supplement: Supplementary file 1 — Supplementary Information file [file 41467_2021_24792_MOESM1_ESM.pdf]

Supplementary informations.

Supplementary Figures 1-7 with legends.

Supplementary Tables 1-5.

Supplementary note: REMARK checklist.

Supplementary Figure 1

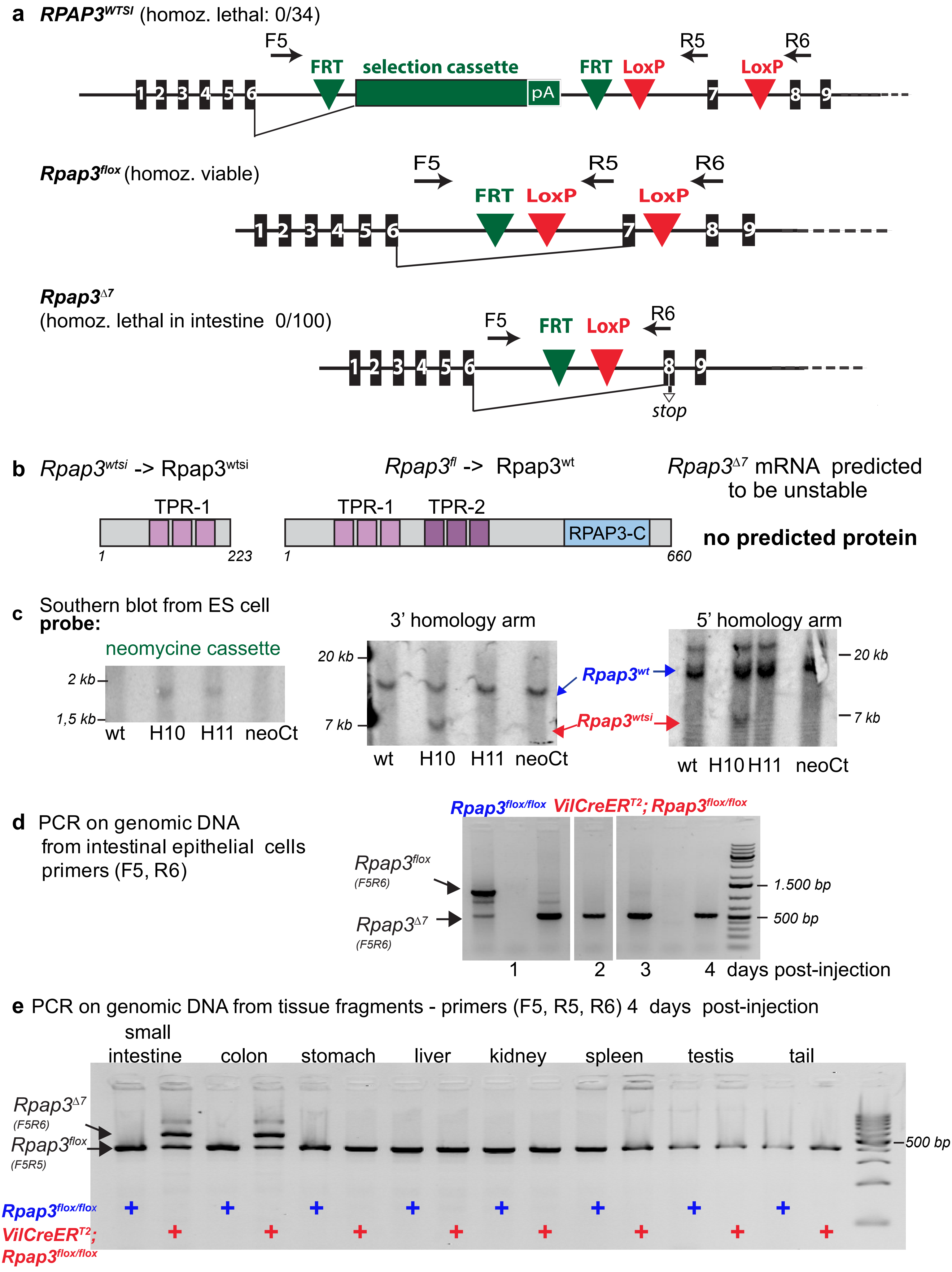

Supplementary Figure 1: Targeting *Rpap3* in the murine intestinal epithelium

(a) Schematic representation of the different *Rpap3* alleles described in this paper. Black boxes represent the 9 first exons in *Rpap3* murine gene with numbering above. Green and red forms represent the inserted elements: green triangles for FLP recombination sequences, green box represents the selection cassette containing the β-galactosidase ORF and the poly-adenylation site (pA), red triangles indicate the *LoxP* sites. Black arrows represent the primers used for genotyping.

(b) Rpap3 proteins encoded by the above-described alleles with domains and amino acid numbering in italic.

(c) Southern blot of genomic DNA showed a correct and unique recombination of the *wtsi* construct at the *Rpap3* locus in the KOMP ES clone H10 but not in H11. Molecular sizes are indicated on the side of the pictures. N=1.

(d) PCR on genomic DNA from small intestine epithelial cells shows that recombination occurs as early as one day after tamoxifen injection in the $VilCreER^{T2}$; $Rpap3^{flox/flox}$ mice, and persists up to 4 days (amplification of a band corresponding to $Rpap3^{\Delta 7}$). PCR with primers F5, R6, on genomic DNA from $Rpap3^{flox/flox}$ mice intestinal epithelial cells amplifies a band corresponding to the floxed, unrecombined *Rpap3* allele (n=1animal/point). Molecular sizes are indicated on the right.

(e) PCR with primers F5, R5, R6 on genomic DNA from organs shows that recombination occurs only in the small intestine and the colon (upper band corresponding to $Rpap3^{\Delta 7}$), but not in the other organs of $VilCreER^{T2}$; $Rpap3^{flox/flox}$ mouse treated with tamoxifen (n=1). Lower band corresponds to unrecombined $Rpap3^{flox}$ allele. Note that extracts were prepared from total small intestine and colon, which comprise epithelium (where recombination takes place) and stroma (which does not express $VilCreER^{T2}$). Molecular sizes are indicated on the right.

Supplementary Figure 2

A. **Olfm4 at day 7**

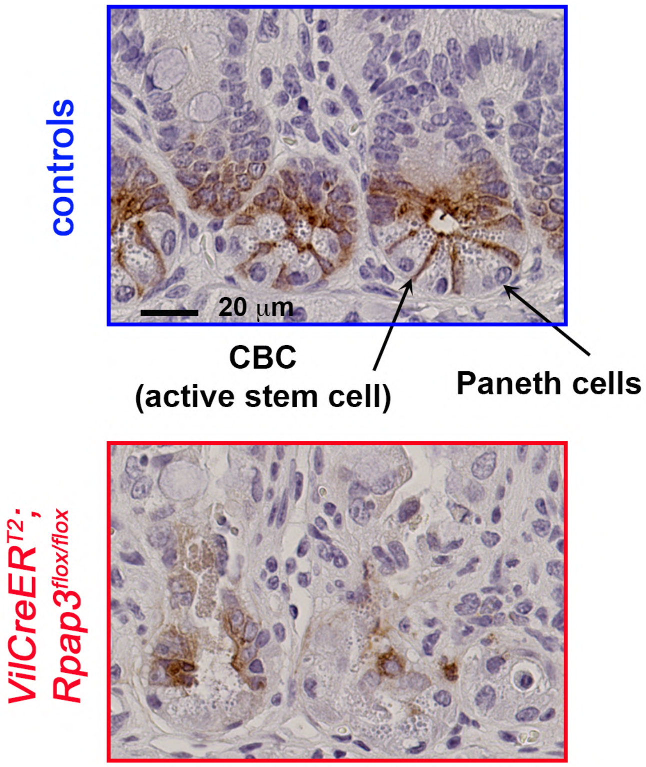

Supplementary Figure 2: **Loss of Olfm4^{+} cells in *Rpap3* KO mice.**

(A) Enlarged fields from the images in Figure 4a revealing Olfm4 staining in crypts of the jejunum from control and *VilCreER^{T2}; Rpap3$^{flox/flox}$* littermates, at day 7, representative of n=5 from two experiments. Scale bar=20 μm is identical for the two pictures.

Supplementary Figure 3

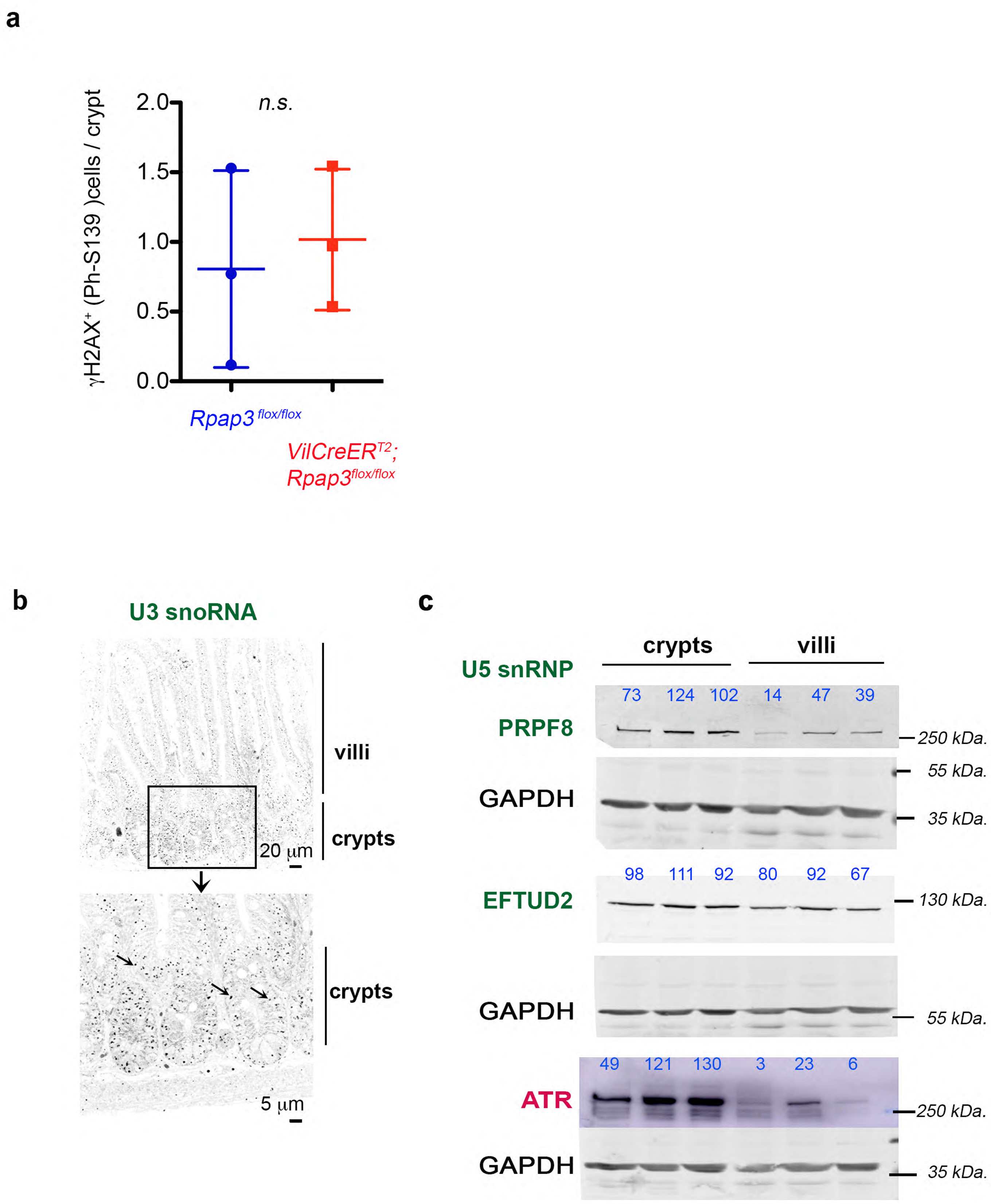

Supplementary Figure 3: **R2TP substrates are concentrated in small intestinal crypts.**

(a) Graph shows quantification of the DNA-damage marker γH2AX in the small intestine from control and *VilCreER^{T2} Rpap3$^{flox/flox}$* mice at day 6 (n=3, one experiment). Mean values with S.D. are indicated for each experimental group.

(b) Micrographs are tissue sections stained for the snoRNA U3, an essential box C/D snoRNA, by FISH, in the crypts from controls animals (top) and inset magnification (bottom), representative for n=4. Scale bars are 20 µm.

(c) Western blot of intestinal epithelial lysates extracted from crypt or villi from 3 control mice are shown. Western blots were probed with anti-PRPF8, EFTUD2, ATR and GAPDH as a loading control. Quantification of the signal ratios are indicated on top of each lane (average for the crypt ratios was arbitrarily set to 100). Molecular weights are indicated on the right.

Supplementary Figure 4

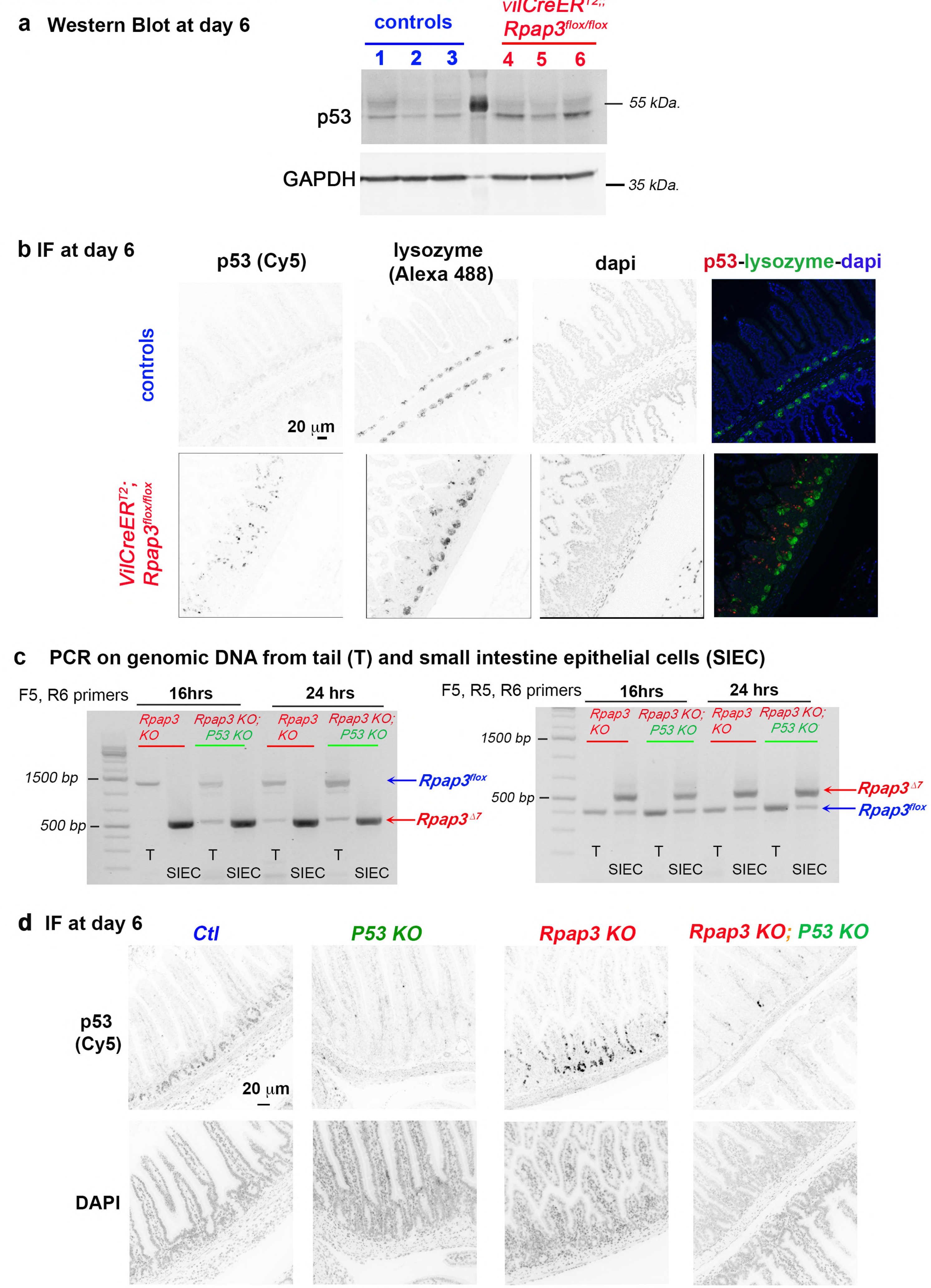

Supplementary Figure 4: R2TP invalidation triggers a p53 checkpoint

(a) Western blot analysis of p53 levels in the lysates of crypt cells from *VilCreER*^{T2}*; Rpap3*$^{flox/flox}$ animals, as compared to control littermates. Each lane represents lysates prepared from one animal with the indicated genotype (1 experiment). GAPDH was used as a loading control. Molecular weights are indicated on the right. Animals belong to a different experiment than those used to monitor p53 by IF, presented in Figure 6a.

(b) Micrographs are tissue sections stained by immunofluorescence for p53 (Cy5, red) and lysozyme, a marker of Paneth cells (Alexa 488, green) in controls (top) and *VilCreER*^{T2}*; Rpap3*$^{flox/flox}$ mice (bottom) at day 6 (n=4). DAPI (blue): nuclei. Scale bar= 20 μm is identical for all pictures.

(c) PCR with primers F5, R6 (left) and F5, R5, R6 (right) on genomic DNA from tails (T) and small intestine epithelial cells (SIEC), extracted from single *Rpap3 KO* (*VilCreER*^{T2}*; Trp53*$^{flox/+}$*; Rpap3*$^{flox/flox}$) or double *Rpap3 KO; P53 KO* (*VilCreER*^{T2}*; Trp53*$^{flox/flox}$*; Rpap3*$^{flox/flox}$) mice, 16 hrs or 24 hrs following tamoxifen injection (n=1). Bands corresponding to unrecombined, *Rpap3*^{flox} and recombined *Rpap3*$^{\Delta 7}$ alleles are indicated by blue and red arrows, respectively (see Supplementary Fig. 1 for a schematic representation of the primers used). Molecular sizes are indicated on the left.

(d) Micrographs are tissue sections stained by immunofluorescence for p53 (Cy5) in control, *P53 KO* (*VilCreER*^{T2}*; Rpap3*$^{flox/+}$*; Trp53*$^{flox/flox}$*), Rpap3 KO (VilCreER*^{T2}*; Rpap3*$^{flox/flox}$*; Trp53*$^{flox/+}$*)* and double *Rpap3 KO; P53 KO (VilCreER*^{T2}*; Rpap3*$^{flox/flox}$*; Trp53*$^{flox/flox}$*)* mice at day 6 (representative of n=3 each single KO, n=7 double KO, from two different experiments). DAPI stained nuclei. Scale bar is shown in control, p53 picture. Note that the faint signal in crypts from control animals is absent in single *P53* and double *RPAP3 P53 KO*s animals.

Supplementary Figure 5

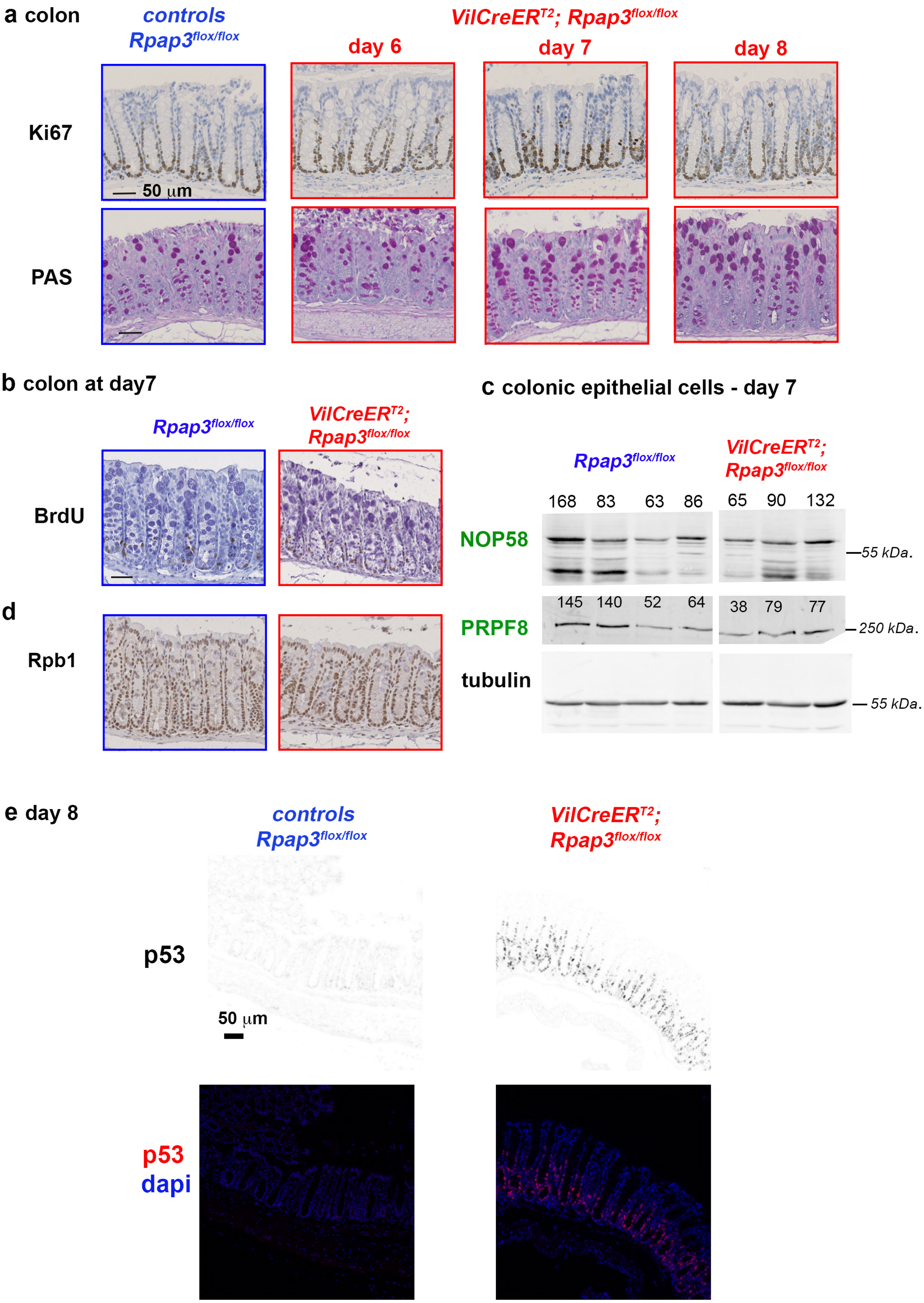

Supplementary Figure 5: R2TP invalidation in the colon

(a) Micrographs are colon sections stained by PAS and IHC for Ki67 in controls *Rpap3*$^{flox/flox}$ (left) and *VilCreER*^{T2}*; Rpap3*$^{flox/flox}$ littermates (right) at day 6, 7, 8 after the first tamoxifen injection. Scale bar= 50 µm is identical for all figures. Each panel is representative of n=8-12 animals from three independent experiments.

(b) IHC for BrdU in controls *Rpap3*$^{flox/flox}$ (left) and *VilCreER*^{T2}*; Rpap3*$^{flox/flox}$ mice (right) at day 7 (n=3). Scale bar= 50 µm is identical as in (A) and (C).

(c) Western blot analysis of preparations enriched for colonic epithelial cells from animals, sacrificed 7 days after the first tamoxifen injection. NOP58, PRPF8 and tubulin were detected with specific antibodies. Quantification of the signal ratios are indicated on top of each lane (average for the control ratios was arbitrarily set to 100). Each lane was loaded with the lysate obtained from one animal of the indicated genotype (observed for n=5 KO animals from 1 experiment). Molecular weights are indicated on the right.

(d) IHC for Rpb1 in controls *Rpap3*$^{flox/flox}$ (left) and *VilCreER*^{T2}*; Rpap3*$^{flox/flox}$ mice (right) at day 7 (n=4). Scale bar is identical as in (A, B).

(e) Micrographs are colon sections stained by IF for p53 in controls *Rpap3*$^{flox/flox}$ (left) and *VilCreER*^{T2}*; Rpap3*$^{flox/flox}$ mice (right) at day 8 (n=3). Scale bar= 50 µm are identical for all pictures.

Supplementary Figure 6

a

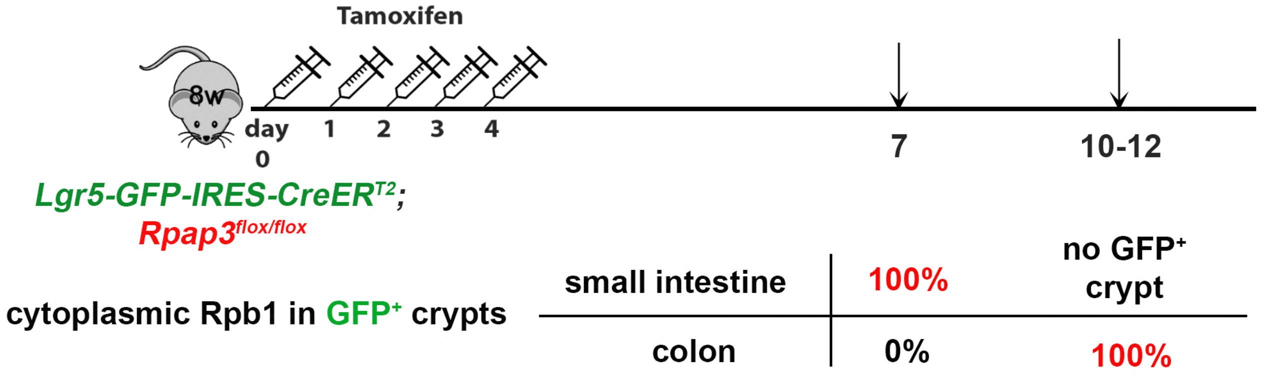

b

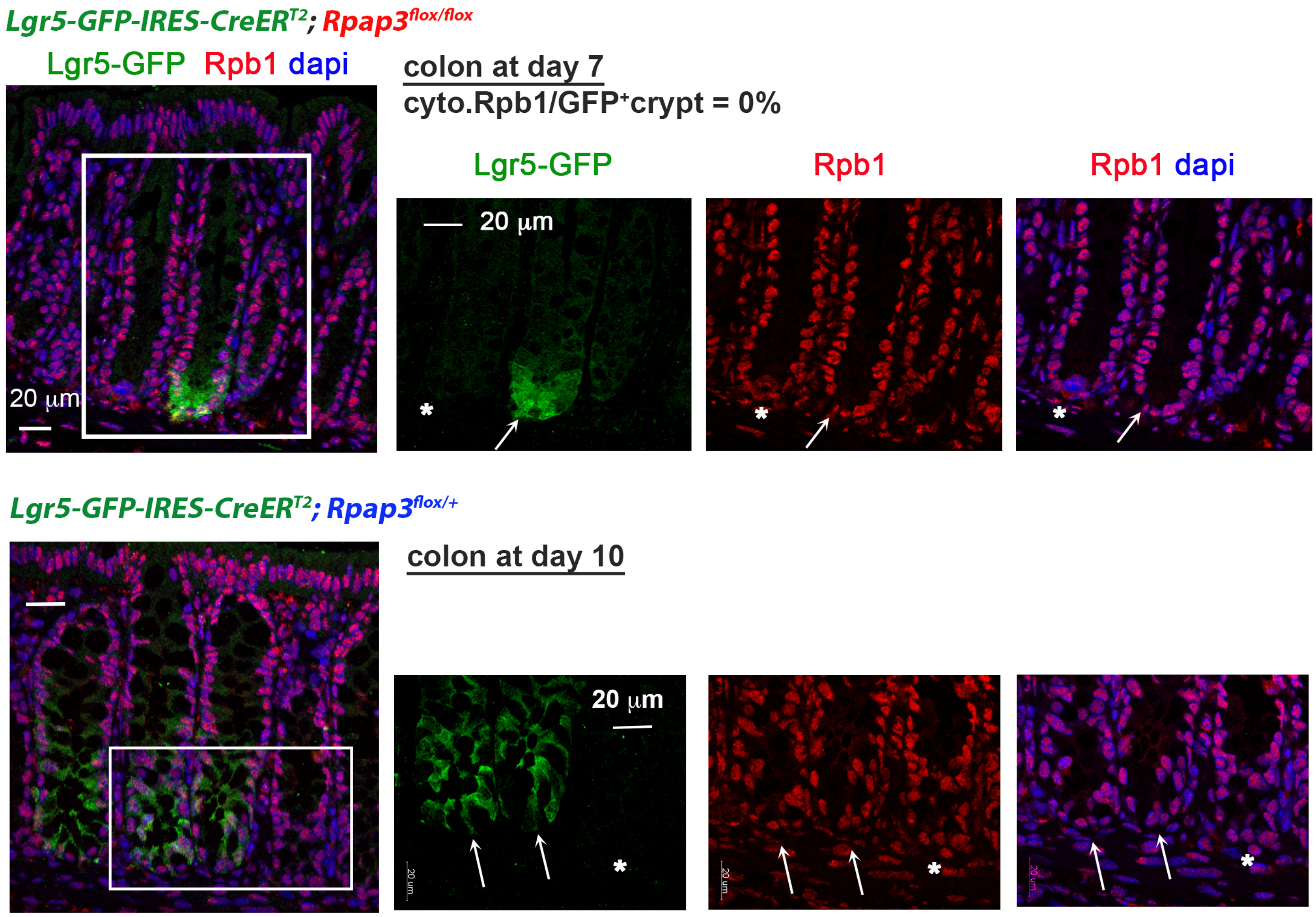

Supplementary Figure 6: Cytoplasmic accumulation of Rpb1 in *Rpap3* KO crypts correlates with cellular turnover.

(a) Experimental setting in the mosaic model *Lgr5-GFP-IRES-CreER*^{T2}*; RPAP3*$^{flox/flox}$. Quantification results of GFP^+ crypts with signal for cytoplasmic Rpb1, as shown in (b). Source data are available as a "Source data file".

(b) Images of tissue sections labelled by immunofluorescence with antibodies against GFP (green) and Rpb1 (red), with DAPI counter-staining of nuclei (blue), representative of n=4-5 animals in two experiments. Please note the mosaic expression of GFP. White arrow: GFP^+ crypts. Asterisks: GFP^- crypts. Scale bars=20 µm are identical for matching panels.

Supplementary Figure 7

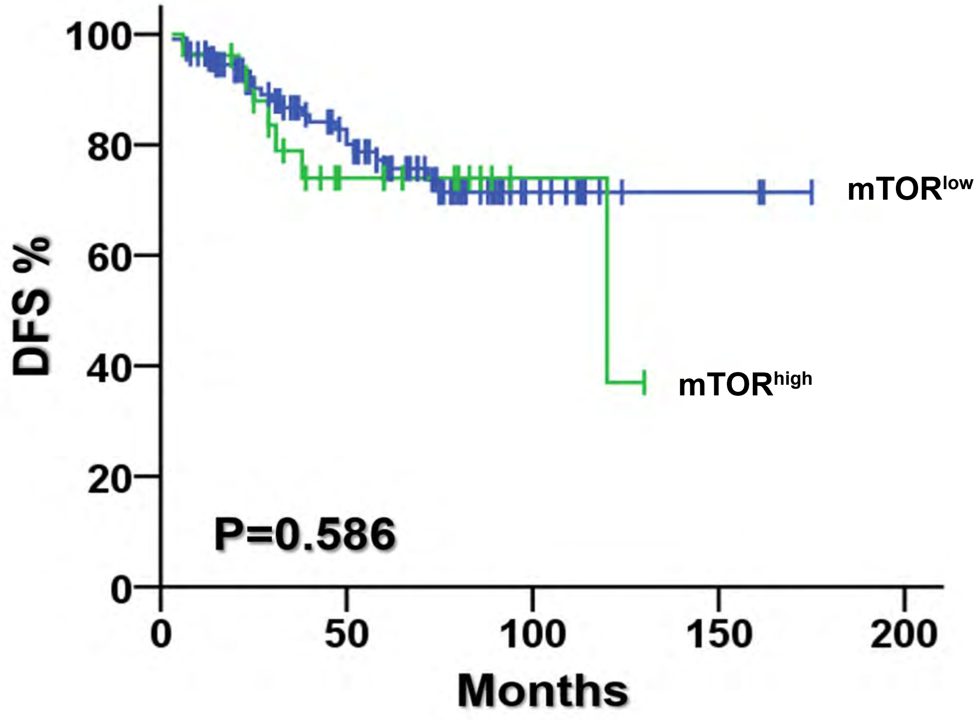

Supplementary Figure 7: Correlation of mTOR expression with patient outcome.

Kaplan-Meier disease-free survival (DFS) analysis among 149 CRC patients according to the expression of mTOR in tumour tissue. The solid green line and dashed blue line indicate CRC cases with $mTOR^{high}$ and TOR^{low}, respectively. Statistical significance was determined by log-rank test (P = 0.586).

Supplementary Table 1. RPAP3 Status According to Clinicopathological Features of CRC Patients (n = 177)

Variable	RPAP3		
	Low: n (%)	High: n (%)	P
Age			
< 60	8 (14.8)	22 (17.9)	
≥ 60	46 (85.2)	101 (82.1)	0.827
Sex			
Male	32 (59.3)	79 (64.2)	
Female	22 (40.7)	44 (35.8)	0.613
Tumor Location			
Colon	49 (90.7)	106 (86.1)	
Rectum	5 (9.3)	17 (13.8)	0.467
Histologic Type			
Adenocarcinoma	37 (68.9)	105 (85.7)	
Mucinous adenocarcinoma	14 (26.7)	12 (9.8)	
Signet-ring cell/undifferentiated carcinoma	3 (4.4)	6 (4.5)	*0.025
Tumor Grade			
1	4 (7.4)	12 (9.8)	
2-3	50 (92.6)	111 (90.2)	0.779
“T” classification			
T1	0 (0.0)	7 (5.7)	
T2	18 (33.3)	55 (44.7)	
T3	35 (64.8)	59 (48.0)	
T4	1 (1.9)	2 (1.6)	0.107
Stage			
I	18 (33.3)	62 (50.4)	
II	36 (66.7)	61 (49.6)	*0.049
Ki-67			
Negative	32 (59.2)	79 (64.2)	
Positive	22 (40.8)	44 (35.8)	0.585
Tumor Relapse			
Absent	48 (88.9)	84 (68.3)	
Present	6 (11.1)	39 (31.7)	*0.003
Patients’ Survival Status			
Alive	49 (90.7)	95 (77.2)	
Dead	5 (9.3)	28 (22.8)	*0.036

* Statistically significant values ($p < 0.05$)

Supplementary Table 2. Multivariate analysis of RPAP3 expression in CRC tumors (n = 177).

Variable	HR	95% CI	P
**Disease-free survival**			
Sex (Male *vs* Female)	1.1	0.6-2.1	0.736
Tumor Location (Rectal *vs* Colon)	1.9	0.9-4.0	0.091
Tumor Grade (2-3 *vs* 1)	1.2	0.4-3.8	0.823
Tumor Stage (II *vs* I)	2.3	1.2-4.5	0.012*
RPAP3 (High *vs* Low)	2.7	1.2-6.5	0.023*
**Overall Survival**			
Sex (Female *vs* Male)	1.1	0.5-2.2	0.885
Tumor Location (Rectal *vs* Colon)	1.4	0.5-3.7	0.520
Tumor Grade (2-3 *vs* 1)	1.2	0.3-5.0	0.835
Tumor Stage (II *vs* I)	2.0	0.9-4.3	0.089
RPAP3 (High *vs* Low)	2.1	0.8-5.5	0.133

*Statistically significant

Supplementary Table 3. Patients and Tumor Characteristics (n = 177)

Variable	Value
Age at	
Median	70.0
Range	36 - 90
Sex	
Male	111
Female	66
Tumor	
Colon	155 (87.6)
Rectal	22 (12.4)
Tumor Stage	
I	80 (45.2)
II	97 (54.8)
Pathological	
Well	16 (9.0)
Moderate	148 (83.7)
Poor	13 (7.3)
RPAP3	
Low	54
High	123

Supplementary Table 4: Antibodies used in IHC or IF on paraffin-embedded tissues.

Rbt=rabbit, M=mouse.

target	Supplier name	catalog number	host species	Clone	Lot	dilution for IHC	dilution for WB
**cleaved caspase-3**	Cell signaling	9664	Rbt	5A1E	45	1/2000	
**Olfm4**	Cell signaling	39141	Rbt	D6Y5A XP	1	1/500	
**ATM**	Cell signaling	2873	Rbt	D2E2	5		1/1000
**ATR**	Cell signaling	13934	Rbt	E1S3S	4		1/1000
**mTOR**	Cell signaling	2972	Rbt		10		1/1000
**p53**	Cell signaling	2524	M	1C12			1/1000
**p53**	Leica	P53-CM5P	Rbt		6062214	1/250	
**Rpb1**	Euromedex	IG-PB-7C2	M	PB-7C2		1/400	
**Biotin BrdU**	Biolegend	339810	M	Bu20a	B185055	1/100	
**TRRAP**	Bertin	G01043	M	2D5			1/1000
**Ki67**	Invitrogen	14-5698-80	rat	SolA15	170719L VA	1/1000	
**GFP**	Invitrogen	A-6455	Rbt		1964399	1/100	

**PIH1D1**	ProteinTech	19427-1-AP	Rbt		0001210 7	1/600	1/1000
**Lysozyme C**	Santa Cruz	sc-27958	goat	C-19	K2315	1/400	
**PRPF8**	Santa Cruz	sc-30207	Rbt	H-300	A303-921A-1		1/200
**human RPAP3**	proprietary	19B11	M			1/10	
**RPAP3**	Sigma	SAB141143 8	Rbt		11312-S4		1/1000
**NOP58**	Sigma	HPA018472	Rbt		A302-719A-M-1		1/100
**α-Tubulin**	Sigma	T6074	M	B-5-1-2	117M48 46V		1/500
**EFTUD2**	Abcam	ab72456	Rbt		A300-957A-1		1/2000
**GAPDH**	Abcam	ab8245	M	6C5	0001578		1/10 000
**γ-H2AX**	Abcam	Ab11174	Rbt		GR2948 90-5	1/20 000	

Supplementary Table 5: Primers.

Name	Sequence
RPAP3-F5	GGTGCCACAGTGTGAGTG
RPAP3-R5	TGCCTCCTGACTCACTACAG
RPAP3-R6	ACCGTGTCGCTACGTCACTGCG
CREERT2-F	TTACGGCGCTAAGGATGACT
CREERT2-R	AAGGCCAGGCTGTTCTTCTT
P53-F	TGC TAG ATG CTT AGG GCT GC
P53-R	GAC TGG CCC TTC TTG GTC TT

	Page no.
**INTRODUCTION**	
We investigated the relationship between RPAP3 expression and the clinical outcome of colorectal cancer (CRC) patients using immunohistochemistry on Tissue Microarrays (TMAs) sections. The purpose was to determine whether RPAP3 expression has any relationship with the outcome of CRC patients. The hypothesis tested was that RPAP3 expression status is a marker of disease-free survival and overall survival in potentially curatively resected CRC.	See last section of **Results**
**MATERIALS AND METHODS**	
Patients	
From 1996 to 2010, 652 patients affected by primary colorectal adenocarcinoma were resected at the Department of Surgery, SS. Annunziata Hospital in Chieti (Italy). Among those referred to the Department of Oncology of the same Hospital, only patients with Stage I and Stage II tumors (n = 190) were tentatively included in the study. Additional inclusion criteria were: (a) informed consent or waiver of consent; (b) age ≥18 years; (c) receipt of at least one follow-up within 5 years. To avoid interaction between response to treatment and RPAP3 expression, only patients not undergoing adjuvant chemotherapy followed up at SS. Annunziata Hospital, and with retrievable archival tumor tissue, were included in the study. Thus, the final number of evaluable patients decreased to 177.	See first section of **Methods**
Specimen characteristics	
CRC tissue samples were fixed in 10% buffered formalin for 24 h, dehydrated and paraffin-embedded. TMAs were constructed by extracting 2-mm diameter cores of histologically confirmed neoplastic invasive CRC areas from each original paraffin block and re-embedding these cores into gridded paraffin blocks, using a precision instrument (MTA, Beecher Instruments, WI). Before performing the immunohistochemical staining, 5 micrometer TMA sections were cut using a microtome and mounted onto histological polarized glass slides.	See first and third sections of **Methods**
Assay methods	
Immunohistochemistry was used to detect the presence of RPAP3 (proprietary 19B11 antibody at 1:10 dilution) and mTOR (Cell Signaling 7C10 antibody; cat. Number #2983, at 1:100 dilution) proteins in primary human CRC specimens. Antigen retrieval was performed by microwave treatment at 750 W (10 min) in 10 mmol/l sodium citrate buffer (pH 6.0). The polymer Envision kit (Agilent) was used for signal amplification. DAB (3,3-Diaminobenzidine) was used as chromogen. The two antibodies used for the immunostainings of CRC human samples have been validated by our previous work (i.e., RPAP3 antibody) and by the manufacturer (i.e., mTOR antibody). The receiver operating characteristic (ROC) curve analysis was applied to obtain the RPAP3 and mTOR positivity thresholds. The maximum Youden index indicated the optimum cut-off: values for RPAP3 and mTOR positive expression were 26% and 10%, respectively. Immunohistochemical analysis was done by a pathologist (RL) who was blinded to the clinical data of the patients.	See last section of **Results,** third sections of **Methods**, Supplementary Table 4 and References 19, 51
Study design	
We retrospectively analyzed 177 assessable CRC samples from patients who received surgical treatment at the SS. Annunziata Hospital in Chieti (Italy) between 1996 and 2010. The median follow-up was 53 months (range 3-238 months).	See last section of **Results**,

Source: McShane LM, Altman DG, Sauerbrei W, Taube SE, Gion M, Clark GM: Reporting recommendations for tumor marker prognostic studies (REMARK). *J Natl Cancer Inst* 2005; 97: 1180-1184.

The primary endpoint was tumor recurrence or death of a patient. Disease-free survival (DFS) was defined as the time from surgery to the first one of the following events: recurrence at local or distant sites or intercurrent death without recurrence. Overall survival (OS) was defined as the interval between the date of surgery and the date of death or the last known follow-up.<br>The association of RPAP3 expression with the outcome, adjusted for other prognostic factors, was tested by Cox's proportional hazards model. The following covariates were included in the multivariate models: gender, tumor location, tumor grade, tumor stage, and RPAP3 status.<br>In our study, during the follow-up period, 25.4% of CRC patients (45 out of 177) had disease relapse, while deaths were observed in 18.6% of CRC patients (33 out of 177). These outweight the 10 to 25 events required to investigate the potential prognostic role of a variable.	first and last sections of **Methods.**
Statistical analysis methods	
The relationships between RPAP3 expression and clinicopathological parameters were investigated by Pearson's χ^2 test. Survival curves were plotted by the Kaplan-Meier method and compared using the log-rank test. The association of RPAP3 expression with the outcome, adjusted for other prognostic factors, was tested by Cox's proportional hazards model.<br>The receiver operating characteristic (ROC) curve analysis was applied to obtain positivity thresholds. The maximum Youden index indicated the optimum cut-off value.	See last sections of **Results** and **Methods**; Supplementary Tables 1 and 2
**RESULTS**	
Data	
Eligible patients were extracted from 652 primary CRC cases consecutively resected between 1996 and 2010 at the Department of Surgery, SS. Annunziata Hospital in Chieti (Italy). Among those referred to the Department of Oncology of the same Hospital, only patients with Stage I and Stage II tumors were tentatively included in the study (n = 190). To avoid interaction between response to treatment and RPAP3 expression, only patients not undergoing adjuvant chemotherapy followed up at SS. Annunziata Hospital, and with retrievable archival tumor tissue, were included in the study. Thus, the final number of evaluable patients decreased to 177. During the follow-up period, 25.4% of CRC patients (45 out of 177) had disease relapse, while deaths were observed in 18.6% of CRC patients (33 out of 177).<br>Patients and tumor characteristics were summarized in Supplemental Table 1, 2 and 3. 157 out of 177 (88.7%) cases expressed RPAP3 in the tumor cell cytoplasm. The proportion of RPAP3-positive cells was in the range of 4-100%, with a mean ± S.E. of 62.6% ± 2.6.	See first section of **Methods**; Supplementary Tables 1 -3
Analysis and presentation	
RPAP3 expression negatively correlated with the tumor stage of CRC (p = 0.049). Compared with stage I tumors, the expression of RPAP3 was decreased in stage II tumors (p = 0.049). Furthermore, compared with mucinous carcinoma, the expression of RPAP3 was increased in CRC of the adenocarcinoma type (p = 0.025). In addition, high RPAP3 expression was positively correlated with the occurrence of tumor relapse (p=0.003) and patients' mortality (p= 0.036).<br>Kaplan-Meier plots comparing patients with RPAP3 expression levels above and below the cut-off value have been reported in Figure 9b. The analysis shows that	See last section of **Results**; Figure 9b; Supplementary Tables 1- 3

patients with RPAP3^high^ tumors have a lower DFS rate than patients with RPAP3^low^ tumors (p= 0.037).<br>Multivariate analysis of DFS adjusted for other prognostic factors shows that RPAP3 expression was a significant prognostic parameter influencing disease relapse (HR = 2.7: 95% CI, 1.2-6.5; p= 0.023), but not the overall survival of patients.	
**DISCUSSION**	
We provide evidence that high RPAP3-expression levels in CRC tissues are associated with poor patient prognosis.	See last section of **Results**

Source: McShane LM, Altman DG, Sauerbrei W, Taube SE, Gion M, Clark GM: Reporting recommendations for tumor marker prognostic studies (REMARK). *J Natl Cancer Inst* 2005; 97: 1180-1184.
