# Supplementary material for: The HSP90/R2TP assembly chaperone promotes cell proliferation in the intestinal epithelium
Source: Nat Commun. 2021 Aug 10;12:4810. doi: 10.1038/s41467-021-24792-4 (PMC8355188; doi:10.1038/s41467-021-24792-4)
Supplement: Supplementary file 3 — Reporting Summary [file 41467_2021_24792_MOESM3_ESM.pdf]

nature research

Corresponding author(s): Berengere PRADET-BALADE

Last updated by author(s): May, 27, 2021

Reporting Summary

Nature Research wishes to improve the reproducibility of the work that we publish. This form provides structure for consistency and transparency in reporting. For further information on Nature Research policies, see our Editorial Policies and the Editorial Policy Checklist.

Statistics

For all statistical analyses, confirm that the following items are present in the figure legend, table legend, main text, or Methods section.

n/a	Confirmed	
☐	☒	The exact sample size (n) for each experimental group/condition, given as a discrete number and unit of measurement
☐	☒	A statement on whether measurements were taken from distinct samples or whether the same sample was measured repeatedly
☐	☒	The statistical test(s) used AND whether they are one- or two-sided<br>*Only common tests should be described solely by name; describe more complex techniques in the Methods section.*
☐	☒	A description of all covariates tested
☒	☐	A description of any assumptions or corrections, such as tests of normality and adjustment for multiple comparisons
☐	☒	A full description of the statistical parameters including central tendency (e.g. means) or other basic estimates (e.g. regression coefficient) AND variation (e.g. standard deviation) or associated estimates of uncertainty (e.g. confidence intervals)
☐	☒	For null hypothesis testing, the test statistic (e.g. F, t, r) with confidence intervals, effect sizes, degrees of freedom and P value noted<br>*Give P values as exact values whenever suitable.*
☒	☐	For Bayesian analysis, information on the choice of priors and Markov chain Monte Carlo settings
☒	☐	For hierarchical and complex designs, identification of the appropriate level for tests and full reporting of outcomes
☒	☐	Estimates of effect sizes (e.g. Cohen's d, Pearson's r), indicating how they were calculated

Our web collection on statistics for biologists contains articles on many of the points above.

Software and code

Policy information about availability of computer code

Data collection	Images were collected using NDP view.2 for Nanozoomer (Hamamatsu), ZEN for Axioscan (Zeiss) or Metamorph for Axioimager Z1 (Zeiss), Leica LAS-AF software for confocal imaging, GE/Amersham Typhoon, Imager 600 and Syngene for WB and DNA gels, respectively.
Data analysis	Data were analyzed using Adobe PhotoshopCS6, Fiji/Image J, Graphpad Prism 5.0, SPSS software 15.0 (SPSS Inc., Chicago, IL).

For manuscripts utilizing custom algorithms or software that are central to the research but not yet described in published literature, software must be made available to editors and reviewers. We strongly encourage code deposition in a community repository (e.g. GitHub). See the Nature Research guidelines for submitting code & software for further information.

Data

Policy information about availability of data

All manuscripts must include a data availability statement. This statement should provide the following information, where applicable:
- Accession codes, unique identifiers, or web links for publicly available datasets
- A list of figures that have associated raw data
- A description of any restrictions on data availability

Transcriptomic analysis of R2TP subunits in human samples (Fig. 9A) used the UCSC Xena platform for public and private cancer genomics data visualization and interpretation https://xenabrowser.net and are available in the source data file.
The datasets concerning the patient biopsies (Fig. 9b, Supplementary Fig. 9) are not publicly available because the database contains sensitive information that could compromise patient privacy but are available. Access to the original clinical records are subject to restrictions due to legal and privacy reasons. It must be authorized by the single patient or patient's legally authorized representative, and a specific request must be issued to:
Direzione Medica / Presidio Ospedaliero "SS. Annunziata"
ASL02 di Lanciano-Vasto-Chieti / Via Dei Vestini / 66100 Chieti (Italy).

Timeframe for response is usually 1 month.
The datasets supporting this study are available in the Article, Supplementary Information, Source Data. Any remaining data that supports the study is available upon reasonable request to pradet@crbm.cnrs.fr.

Field-specific reporting

Please select the one below that is the best fit for your research. If you are not sure, read the appropriate sections before making your selection.

- [x] Life sciences
- [ ] Behavioural & social sciences
- [ ] Ecological, evolutionary & environmental sciences

For a reference copy of the document with all sections, see nature.com/documents/nr-reporting-summary-flat.pdf

Life sciences study design

All studies must disclose on these points even when the disclosure is negative.

Sample size	Sample sizes were not predetermined and were chosen dependent on animal availability. All experiments were performed independently, with n>3 controls and KO mice (n>3 for each group, each time point, in each experiment). Observations were conducted on animals of at least two independent experiments. Sample size is described for each experiment. Statistical analysis confirmed the pertinence of the sample size.
Data exclusions	The described phenotypes systematically followed the same kinetics, wether in males or females. We did not exclude any data nor animal.
Replication	We performed 8 independent experiments on VilCreERT2; Rpap3flox/flox mice, 5 on Lgr5-GFP-CreERT2; Rpap3 flox/flox mice and two on VilCreERT2 Trp53flox/flox Rpap3 flox/flox involving each several time-points and n>3 KO animals per point (males or females). Tissue slides were systematically stained for HE and Ki67, and PAS for colons, minimally. Phenotypes were 100% penetrant, as we observed total reproducibility. For Western blotting and IF, images are representative of at least 3 animals per genotype, as indicated in the corresponding figure legends.
Randomization	Experiments were conducted with animals of the same age (8 -10 weeks), obtained from different crosses, with each control or KO group mixing sex and littermates in each group to increase randomization. Mice were allocated to control or KO group considering their genotype. Such homogenization rendered covariates analysis irrelevant to these experiments.<br>Covariates for mouse weight variations was analysed by two-way ANOVA in Fig. 1e as described in the figure legend.<br>For studies on human data, group allocation was performed after blinded tissue analysis, as described in the Results and Methods sections. Supplementary tables 1-3 provide informations and caracteristics about the dataset with analysis of covariates.
Blinding	All histological results (from murine or human samples) were blinded to the observers, with the genotype unknown. Each slide was analysed by at least two independent, blinded observers.

Reporting for specific materials, systems and methods

We require information from authors about some types of materials, experimental systems and methods used in many studies. Here, indicate whether each material, system or method listed is relevant to your study. If you are not sure if a list item applies to your research, read the appropriate section before selecting a response.

Materials & experimental systems

n/a	Involved in the study	
[ ]	[x]	Antibodies
[x]	[ ]	Eukaryotic cell lines
[x]	[ ]	Palaeontology and archaeology
[ ]	[x]	Animals and other organisms
[ ]	[x]	Human research participants
[x]	[ ]	Clinical data
[x]	[ ]	Dual use research of concern

Methods

n/a	Involved in the study	
[x]	[ ]	ChIP-seq
[x]	[ ]	Flow cytometry
[x]	[ ]	MRI-based neuroimaging

Antibodies

Antibodies used	Antibodies used in the study are all listed in Methods sections and Supplementary Table 4 provides information about supplier, reference&lot number, host species and concentration used. Additional information is available upon request.<br>Cleaved caspase-3 Cell signaling 9664 Rbt 5A1E 45 1/2000<br>Olfm4 Cell signaling 39141 Rbt D6Y5A XP 1 1/500<br>ATM Cell signaling 2873 Rbt D2E2 5 1/1000<br>ATR Cell signaling 13934 Rbt E1S3S 4 1/1000<br>mTOR Cell signaling 2972 Rbt 10 1/1000

p53 Cell signaling 2524 M 1C12 1/1000
p53 Leica P53-CM5P Rbt 6062214 1/250
Rpb1 Euromedex IG-PB-7C2 M PB-7C2 1/400
Biotin BrdU Biolegend 339810 M Bu20a B185055 1/100
TRRAP Bertin G01043 M 2D5 1/1000
Ki-67 Invitrogen 14-5698-80 rat SolA15 170719LVA 1/1000
GFP Invitrogen A-6455 Rbt 1964399 1/100
PIH1D1 ProteinTech 19427-1-AP Rbt 00012107 1/600 1/1000
Lysozyme C Santa Cruz sc-27958 goat C-19 K2315 1/400
PRP8 Santa Cruz sc-30207 Rbt H-300 A303-921A-1 1/200
human RPAP3 proprietary 19B11 M 1/10
RPAP3 Sigma SAB1411438 Rbt 11312-S4 1/1000
NOP58 Sigma HPA018472 Rbt A302-719A-M-1 1/100
α-Tubulin Sigma T6074 M B-5-1-2 117M4846V 1/500
EFTUD2 Abcam ab72456 Rbt A300-957A-1 1/2000
GAPDH Abcam ab8245 M 6C5 0001578 1/10 000-H2AX
Abcam Ab11174 Rbt GR294890-5 1/20 000

Validation

All antibodies have been validated by the manufacturers, as well as by independent groups which reported their use in several publications, notably in the CiteAb platform (https://www.citeab.com/). RHEM histology facility and our study herein provide additional validations for use of the anti-p53, -cleaved caspase 3, -ki67 -Olfm4 - GFP, -lysozyme C, and BrdU in HIC/IF. Anti-Rpb1 was validated for IF/IHS in a previous study (Boulon et al., Mol Cell, 2010). Anti-Pih1d1 was validated in WB on murine samples and its usage in IHC was validated herein (Fig. 1c).
For antibodies usage in WB, the size of the observed bands were always verified with published data. This is especially relevant for protein such as PRP8, ATM, ATR, mTOR and TRRAP, which have caracteristic high molecular weights (>250 kDa). Anti-TRRAP antibody has been validated for WB by our group using proprietary cell lines with degradable TRRAP.
19B11 is a customary antibody that has been validated by us (WB and IF on human cell lines) and published previously (Machado-Pinilla, 2012).

Animals and other organisms

Policy information about studies involving animals; ARRIVE guidelines recommended for reporting animal research

Laboratory animals

Mice strains: Rpap3<tm1a(KOMP)Wtsi> ; Tg(CAG-flpo)1Afst, Tg(Vil1-cre)20Syr, Tg(Vil1-cre/ERT2)23Syr, Lgr5tm1<cre/ERT2>Cle and Trp53<tm1Brn> were used to create VilCre Rpap3 flox/+; VilCreERT2 Rpap3 flox/flox; Lgr5-GFP-IRES-CreERT2 Rpap3 flox/flox; VilCreERT2 Trp53 flox/flox Rpap3 flox/flox lines. Mice were housed in temperature-controlled ventilated cages (20-22°C) with a 12h light-dark cycle, with percentage of humidity between 45 and 55%, and maintained in pathogen-free conditions in the institute animal facility, in strict accordance with the ARRIVE guidelines. Male and females were used at age 8-10 weeks for experimentations.

Wild animals

No wild animal was used in this study.

Field-collected samples

This study did not involve any field-collected sample.

Ethics oversight

Mouse experiments were performed in strict accordance with the guidelines of the European Community (86/609/EEC) and the French National Committee (87/848) for care and use of laboratory animals, comply the ARRIVE guidelines and were approved by the French Ministry of Higher Education, Research and Innovation (reference APAFIS#18685) to be performed in the institute animal facility (agreement # F3417216). This is indicated in the methods section.

Note that full information on the approval of the study protocol must also be provided in the manuscript.

Human research participants

Policy information about studies involving human research participants

Population characteristics

The study cohort included 111 (63%) males and 66 (37%) females. The median age of included patients was 70 years (range 36-90). Primary tumors (colorectal adenocarcinoma) were located in the colon in 155 (87%) cases and in the rectum in 22 (13%). The clinicopathological classification and the stage were determined
according to the American Joint Committee on Cancer (AJCC) TNM staging system (AJCC Cancer Staging Manual (8th ed.). There were 80 stage I and 97 stage II tumors. Tumor grade was classified as: well-differentiated (G1) in 16 (9%), moderately differentiated (G2) in 148 (84%), and poorly differentiated (G3) in 13 (7%) cases.

Recruitment

From 1996 to 2010, 652 patients affected by colorectal adenocarcinoma were resected at the Department of Surgery, SS. Annunziata Hospital in Chieti. Among those referred to the Department of Oncology of the same Hospital only patients with Stage I and Stage II tumors (n = 190) were tentatively included into the study. To avoid interaction between response to treatment and RPAP3 expression, only patients not undergoing adjuvant chemotherapy, followed-up at our institution, and with retrievable archival tumor tissue were included into the study. Thus, the final number of evaluable patients decreased to 177, mainly due to unavailability of tumor tissue.
The above reported eligibility criteria make selection bias unlikely. Since most of the patients were excluded due to unavailability of archival tissue sample, it is unlikely that the relationship between outcome and expression of RPAP3 is different for included and excluded patients. Finally, all major confounders were accounted for (patients age, stage, histology,

	tumor grade).
Ethics oversight	This series has been already reported (Piccolo et al.). Written informed consent was obtained from all patients. A study protocol for the use of this series for prognostic studies was approved by the local Institutional Research Ethics Committee Comitato Etico delle Province di Chieti e Pescara e dell' Università degli studi "G. D'Annunzio" di Chieti e Pescara, and conducted following REMARK guidelines.

Note that full information on the approval of the study protocol must also be provided in the manuscript.
